# A prediction model for bacteremia and transfer to intensive care in pediatric and adolescent cancer patients with febrile neutropenia

**DOI:** 10.1038/s41598-022-11576-z

**Published:** 2022-05-06

**Authors:** Muayad Alali, Anoop Mayampurath, Yangyang Dai, Allison H. Bartlett

**Affiliations:** 1 Department of Pediatrics, Division of Infectious Diseases, University of Chicago Medicine, Chicago, IL USA; 2 Department of Pediatrics, The University of Chicago, Chicago, IL USA; 3 Center for Research Informatics, The University of Chicago, Chicago, IL USA

**Keywords:** Haematological cancer, Paediatric cancer

## Abstract

Febrile neutropenia (FN) is a common condition in children receiving chemotherapy. Our goal in this study was to develop a model for predicting blood stream infection (BSI) and transfer to intensive care (TIC) at time of presentation in pediatric cancer patients with FN. We conducted an observational cohort analysis of pediatric and adolescent cancer patients younger than 24 years admitted for fever and chemotherapy-induced neutropenia over a 7-year period. We excluded stem cell transplant recipients who developed FN after transplant and febrile non-neutropenic episodes. The primary outcome was onset of BSI, as determined by positive blood culture within 7 days of onset of FN. The secondary outcome was transfer to intensive care (TIC) within 14 days of FN onset. Predictor variables include demographics, clinical, and laboratory measures on initial presentation for FN. Data were divided into independent derivation (2009–2014) and prospective validation (2015–2016) cohorts. Prediction models were built for both outcomes using logistic regression and random forest and compared with Hakim model. Performance was assessed using area under the receiver operating characteristic curve (AUC) metrics. A total of 505 FN episodes (FNEs) were identified in 230 patients. BSI was diagnosed in 106 (21%) and TIC occurred in 56 (10.6%) episodes. The most common oncologic diagnosis with FN was acute lymphoblastic leukemia (ALL), and the highest rate of BSI was in patients with AML. Patients who had BSI had higher maximum temperature, higher rates of prior BSI and higher incidence of hypotension at time of presentation compared with patients who did not have BSI. FN patients who were transferred to the intensive care (TIC) had higher temperature and higher incidence of hypotension at presentation compared to FN patients who didn’t have TIC. We compared 3 models: (1) random forest (2) logistic regression and (3) Hakim model. The areas under the curve for BSI prediction were (0.79, 0.65, and 0.64, *P* < 0.05) for models 1, 2, and 3, respectively. And for TIC prediction were (0.88, 0.76, and 0.65, *P* < 0.05) respectively. The random forest model demonstrated higher accuracy in predicting BSI and TIC and showed a negative predictive value (NPV) of 0.91 and 0.97 for BSI and TIC respectively at the best cutoff point as determined by Youden’s Index. Likelihood ratios (LRs) (post-test probability) for RF model have potential utility of identifying low risk for BSI and TIC (0.24 and 0.12) and high-risk patients (3.5 and 6.8) respectively. Our prediction model has a very good diagnostic performance in clinical practices for both BSI and TIC in FN patients at the time of presentation. The model can be used to identify a group of individuals at low risk for BSI who may benefit from early discharge and reduced length of stay, also it can identify FN patients at high risk of complications who might benefit from more intensive therapies at presentation.

## Introduction

Febrile neutropenia (FN) is a common condition in children receiving chemotherapy and can be life-threatening^1,2^. An evidence-based guideline grounded in an understanding of which clinical characteristics and laboratory tests most accurately predict FN patients at high risk for severe illness could prompt more aggressive management and intensive monitoring. Conversely, it could help identify patients at low, or no, risk of serious clinical infection who may benefit from early discharge and less parenteral antibiotics during FN admission^3–5^.

Several prediction rules based on clinical and laboratory parameters have been developed^6–9^ for determining which FN patients are at risk for complications. However, these clinical decisions rules (CDRs) vary across populations and geographical locations, making it difficult to develop a single set of criteria to be used in clinical settings^10,11^. The available risk model studies have several limitations, including small study populations lacking independent validation, frequent missing values, and differences in the predictive factors considered. These CDRs have looked to predict severe sepsis, bacteremia, documented infection^12^, or need for critical care^12–14^ as outcomes.

To overcome the limitations of previous studies, efforts are under way to develop and validate risk models based on large studies in representative pediatric populations of patients receiving systemic chemotherapy. PICNICC (Predicting Infectious Complications in Children with Cancer) model was published as a mean of predicting complication in pediatric oncology patients^15^. This study was limited by its reliance on study-defined microbiologically documented infection (MDI) as the endpoint outcome, rather than a more comprehensive, patient-centered assessment of adverse outcomes such as transfer to intensive care. Additionally, MDI represent a large spectrum of severity of infections, which could range from mild skin infection to severe invasive infection. A recent study found that PICNICC risk stratification schema performed poorly in adolescents/young adults with cancer^16^. There are few studies of prediction models in pediatric cancer patients with FN in the USA. Hakim et al.^17^ from St. Jude hospital in Memphis, Tennessee, developed a model for predicting severe infections and adverse outcomes in FN based on a large sample size of pediatric cancer patients at St. Jude Children's Research Hospital. Authors in this study found initial clinical impression using subjective variable such as “sick appearance” as independent risk factor in their prediction model.

The aims of this study are to examine performance of Hakim et al. model in our FN cohort. We try also to define relevant variables at the time of FN presentation and develop and validate a risk prediction model for bloodstream infection (BSI) and transfer to intensive care unit (TIC) in pediatric and adolescent cancer patients. Both prediction models were built for both outcomes using logistic regression and random forest and compared with Hakim model.

## Methods

### Setting and study population

A retrospective cohort study was conducted at University of Chicago Medicine (UCM) Comer Children’s Hospital, a 172-bed acute care hospital located on Chicago’s south side that serves a diverse pediatric population. The medical center offers highly specialized cancer care, including stem cell transplant (SCT)^18^.

**Figure 1:**
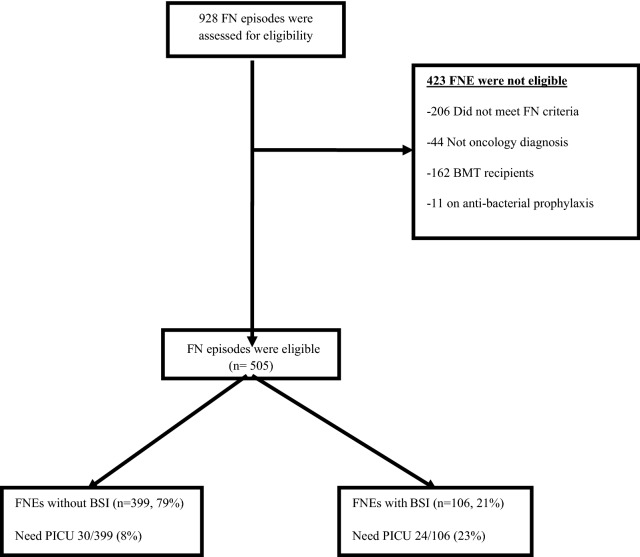
Study flow diagram. Febrile neutropenia (FN) with and without BSI and need to PICU. *FNE* Febrile neutropenia episodes, *BSI* blood stream infection. *PICU* pediatric intensive care, *BMT* bone marrow transplant.

Study protocols were approved by the Clinical Trials Review Committee and the University of Chicago Institutional Review Board. We confirm all methods were carried out in accordance with relevant guidelines and regulations. The need for informed consent was formally waived by the approving committee. To identify appropriate patients for inclusion, the Clinical Research Data Warehouse, operated by the Center for Research Informatics, was queried for hospital admissions of patients 24 years of age or younger admitted to Comer Children’s Hospital from March 2009 to December 2016 for discrete clinical and laboratory values as well as diagnosis codes using International Classification of Disease, 9th Revision, Clinical Modification (ICD-9-CM) codes (ICD-10 after October 2015) to identify patients with FNE. Oncology patients were identified with ICD codes for malignancy or SCT diagnoses. Neutropenic patients were identified by ICD code for neutropenia OR absolute neutrophil count (ANC) < 500. Febrile patients were identified by temperature ≥ 38.0 °C (≥ 100.4℉) in a 24-h period (Fig. 1). A list of predictor variables is provided in Supplementary Table 2.

Manual electronic health record (EHR) review was conducted to verify that FN episodes were appropriate for inclusion based on the above characteristics. All episodes not meeting the above-mentioned criteria, including febrile non-neutropenic episodes, were excluded. SCT recipients who develop FN after transplant (Day #0) were excluded. For patients with more than one admission for FN, each admission was counted as a separate episode. Data for all variables listed below were collected during manual EHR review.

#### Data collection

Study data were collected by EHR review and managed using RED Cap^19^. The risk prediction variables are a mixture of clinical findings, and basic laboratory studies, which are readily available at admission or at early reassessment, and which are reproducible across all other settings. Variables collected include: patient demographics (age, gender), oncologic diagnosis, history of BSI; clinical features (blood pressure, documented complaints [gastrointestinal symptoms, mucositis, upper respiratory symptoms and chills], and location at presentation); and laboratory data at presentation (white blood cell [WBC] count, absolute neutrophil count [ANC], absolute monocyte count [AMC], absolute lymphocyte count [ALC], platelet count, hemoglobin, duration of neutropenia before FN episode, and blood culture results.). For temperature, we report the maximum temperature between presentation and antibiotic initiation. To decrease observer bias, risk factors and outcomes of interest were recorded by two investigators in order to blind associated variables in each FNE. We did not evaluate other potential adverse events besides BSI or transfer to ICU (e.g., culture negative sepsis) so we could focus on discrete FN-related outcomes.

#### Outcome and predictor variables

The primary outcome of BSI was defined by a positive blood culture (within 7 days from the date of FN onset) with a pathogenic organism or with a contaminant (determined by the National Health Safety Network (NHSN) criteria for skin commensals) that the clinical team decided to treat as a pathogen^2^. The secondary outcome of TIC was defined by transfer to ICU within 14 days from the date of FN onset which is defined as the first timepoint with both documented fever and documented neutropenia regardless of patient location. A list of predictor variables is provided in Supplementary Table 1. Fever was defined as a single oral temperature of > 38.3ºC or temperature of > 38.0ºC sustained over a 1-h period or on more than one occasion in a 24-h period. If there were multiple temperature readings, we reported the maximum temperature between first symptoms and antibiotic initiation^2^. Neutropenia was defined as ANC < 500/mm^3^. Hypotension was defined as a systolic blood pressure < 5^th^ percentile for age and sex^20^. Each FN episode was independently associated with outcomes using a variable indicating the number of prior FN episodes (categorized as 0, 1 and > 1) in order to control for recurrent episodes. We characterized underlying oncologic conditions in patients as belonging to one of four groups based on intensity of chemotherapy: (1) mixed leukemia and acute myeloid leukemia (AML); (2) acute lymphoblastic leukemia (ALL) and lymphoma (Hodgkin’s and non-Hodgkin’s); (3) neuroblastoma (NB); and (4) all other solid tumors.

#### Clinical patient management

Pediatric FN patients were managed per standard institutional practice, which did not undergo any major changes during the study period. Ceftazidime is an initial empiric antimicrobial for FN patients with vancomycin ± gentamicin added based on clinical presentation (i.e., concern for central venous catheter infection or septic shock). Cefepime was administered instead of ceftazidime for selected patients with high-risk FN such as acute myelogenous leukemia (AML). Empiric antifungal therapy (usually liposomal amphotericin B) was added if the patient remained febrile on day 5 of antibiotics and if neutropenia was expected to last longer than 5 to 7 days. Antibacterial prophylaxis was not routinely used; the small number of patients who did receive prophylaxis were excluded.

### Prediction model

We utilized two machine learning techniques, logistic regression and random forest (RF), to predict BSI and TIC based on variables collected at time of FN presentation. While logistic regression models the association between the outcome and predictors in linear terms, RFs explore complex non-linear relationships between variables to further improve prediction accuracy. We split our dataset longitudinally into model derivation (years 2009–2014, *n* = 343 [68%]) and independent prospective validation (years 2015–2016, *n* = 162[32%]) cohorts, this step allows us to determine model performance if implemented in our hospital at the start of the validation period, providing a better real-world assessment of performance in comparison to a random derivation-validation data split. Hyper-parameter optimization for random forests was performed on the derivation cohort using fivefold cross validation. Final predictions were performed on the prospective validation dataset and the area under the receiver operating characteristic curve (AUC) was compared between the logistic regression model (LReg) the RF model, and Hakim et al.^17^ model for both BSI and TIC. Variable importance plots were created to determine the variables most crucial to the prediction of each outcome using the RF model. Analyses were performed using R version 3.3 (R Project for Statistical Computing), with two-sided *P* < 0.05 denoting statistical significance. We had no variables with > 10% missing values. For variables with < 10% missing values, we used imputation. Mixed-effects logistic regression models were fit for the PICU, and BSI outcomes with the inclusion of a random subject effect to account for the multiple observations per patient.

## Results

### Study population

A total of 505 FN episodes (FNEs) were identified in 230 patients. FN episodes during a unique admission ranged from 1 to 3 episodes with complete ANC recovery between these episodes.

The median age was 11.5 (SD = 5.5) years and 47% were female (Table 1). Among 505 FNEs, 106 (21%) developed BSI (Table 1). The most frequent underlying diagnosis was ALL/lymphoma. Rates of BSI were highest among patients with AML (Table 1). The majority of FN cases 420 (83%) were either admitted directly from the emergency department (ED) or from clinic.

**Table 1:** Characteristics of FN episodes among pediatric cancer (*N* = 505).

	All episodes (*n* = 505)	FN episodes with positive blood culture (*n* = 106)	FN episodes without positive blood culture (*n* = 399)	OR (CI 95%)	*P*-value
Age, median (SD)	11.5 (5.5)	12 (6.8)	10.7 (6.1)		0.7
Female sex, *n* (%)	107 (47)	36 (48)	71 (46)		0.8
Cancer diagnosis, *n* (%)					
ALL/lymphoma	218(43%)	32 (30%)	186 (46%)		0.09
AML/mixed leukemia	89(18%)	37 (35%)	52 (13%)	**2.8 (1.25–6.36)**	** < 0.01**
Neuroblastoma	69(14%)	22 (21%)	47 (13%)		0.08
Other solid tumors	129(25%)	15(14%)	114 (28%)		0.07
Neutropenia > 7 days prior FN, *n* (%)					
Yes	346 (68)	69 (65)	277 (69)		0.8
No	105 (21)	33 (31)	72 (18)		
Unknown	54 (11)	4 (4)	50 (13)		
Prior BSI, *n* (%)					
Yes	133 (26)	44 (42)*	89 (22)	**2.4 (1.12–5.35)**	** < 0.01**
No	372 (74)	62 (58)	310 (78)		
GI symptoms*** *n* (%)					
Yes	173 (34)	36 (34)	137 (34)		0.9
No	332 (66)	70 (64)	263 (66)		
Mucositis *n* (%)					
Yes	111(22)	23 (21)	88 (22)		0.9
No	394 (78)	83(79)	312 (78)		
VURI**					
Yes	167 (33)	19 (18)*	148 (37)		0.03
No	268 (53)	72 (68)	196 (49)		
Unknown	70 (14)	28 (26)	58 (14)		
Chills					
Yes	28 (6)	13 (12)*	15 (4)	**2.22(1.40- 4.56)**	**0.02**
No	477 (94)	93 (88)	384 (96)		
Hypotension					
Yes	89 (18)	39 (36)*	50 (12.5)	**3.15 (1.30–8.04)**	** < 0.01**
No	416 (82)	75 (70)	341 (86)		
Temperature height at presentation					
> = 39	122 (25)	41 (39)*	81 (20)	**3.09 (1.59–7.16)**	** < 0.01**
< 39	383 (75)	65 (61)	318 (80)		
Chemotherapy in last 2 weeks					
Yes	282 (76)	78(74)	303 (76)		0.7
No	124(34)	28 (26)	96 (24)		
Inpatient (location FN)					
Yes	85 (17)	27 (25)*	59 (15)	**1.7 (1.03–2.98)**	**0.02**
No	420 (83)	79 (75)	341 (85)		
Hx FN > 1					
Yes (2,3 or 4 episodes)	311 (61)	81 (76)*	230 (57)	1.6 (1.02–3.45)	**0.04**
No (0 or 1 episode)	194 (39)	29 (27)	165 (41)		
ALC, *n* (%)					
< 300	156 (31)	44 (41)*	112 (28)	**1.8 (1.08–4.83)**	**0.02**
> 300	342 (68)	61 (58)	281 (70)		
Unknown	7 (1)	1 (1)	6 (2)		
ANC, *n* (%)					
< 100	362 (72)	78 (74)	284 (71)		0.713
> 100	143 (28)	28 (26)	115 (29)		
AMC *n* (%)					
< 100	389 (77)	84 (79)	304 (76)		0.68
> 100	105 (21)	19 (18)	86 (22)		
Unknown	12 (2)	3 (3)	9 (2)		
Platelet					
< 50	308 (61)	226 (57)*	82 (77)	**2.1 (1.13–5.72)**	**0.01**
> = 50	197 (39)	173 (43)	24 (23)		
Hb < 7					
Yes	103 (20)	29 (27)	74 (18)		0.07
No	402 (80)	77 (73)	325 (82)		
Prior GCSF					
Yes	66 (13)	57 (14)	9 (9)		0.158
No	439 (87)	342 (86)	97 (91)		
Admitted to the PICU, *n* (%)	54 (11)	24 (23)*	30 (8)	**4.5 (2.2–8.7)**	** < 0.01**

*P < 0.05 compared to patient admission that did not develop positive blood culture, ** VURI viral upper respiratory infection documented by RVP , ***includes subjective symptoms such as vomiting, diarrhea, or abdomen pain.

In bivariable analysis, FNEs that resulted in BSI, compared with FNEs that did not result in BSI, were more likely to have underlying diagnosis of AML; history of a prior BSI; thrombocytopenia < 50 × 10^9^/L; ALC < 300/mm^3^; hypotension; higher fever; chills, and inpatient status at the time of FNE (Table 1). A total of 115 organisms were recovered during 106 episodes of BSI in 77 patients. Gram-positive bacteremia was detected in 65/106 (56.5%) and Gram-negative bacteremia was detected in 46/106 (43.3%) episodes. Polymicrobial bacteremia was detected in 7/106 (6.6%).

TIC rate was 54/505 (10.6%), though the majority of TIC occurred in the first week of FN onset 46/54 (85%), but still 8/54 (15%) had clinical worsening after day 7–14 require admission in PICU.

**Figure 2:**
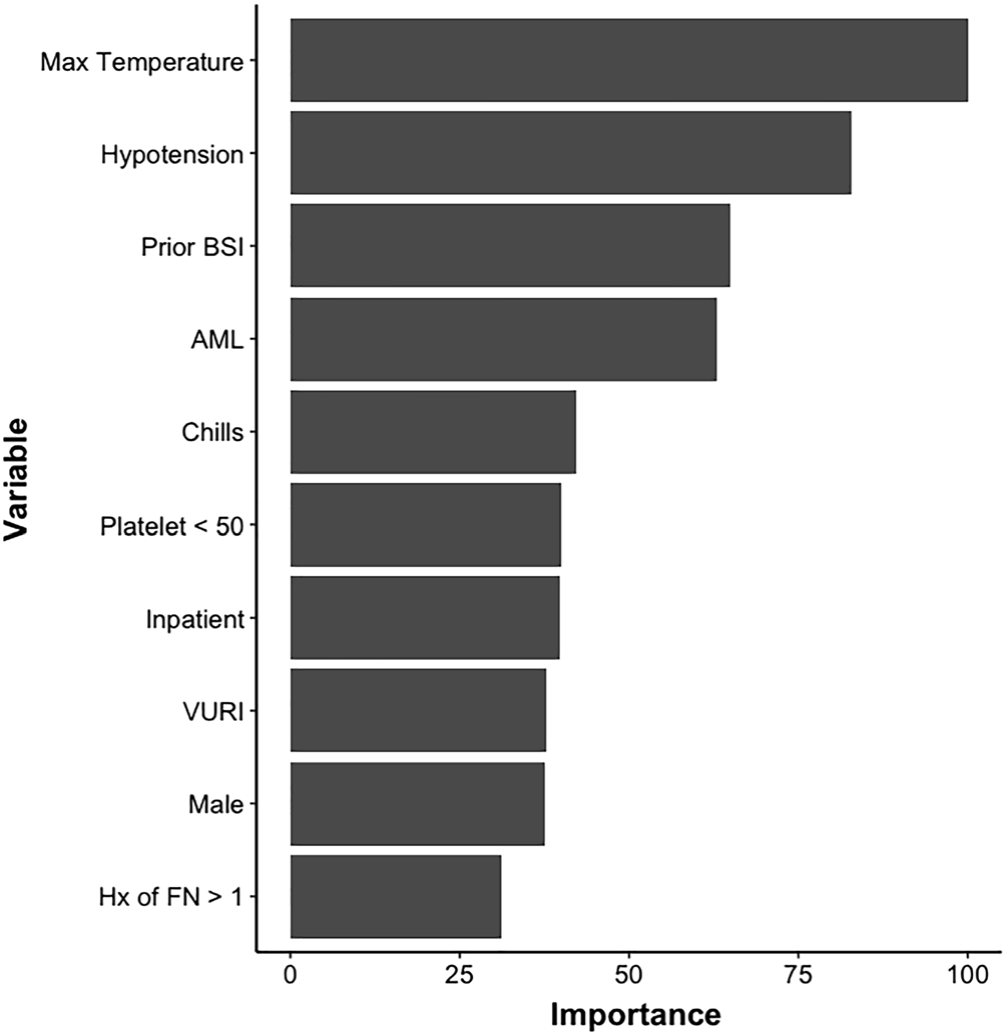
Depicts a list of variables important to the prediction of bactremia using the Random Forest model. Temperature height and hypotension recorded at time of presentation of FN episode, prior positive blood culture, and AML diagnosis were variables that contributed the most to BSI prediction.

**Figure 3:**
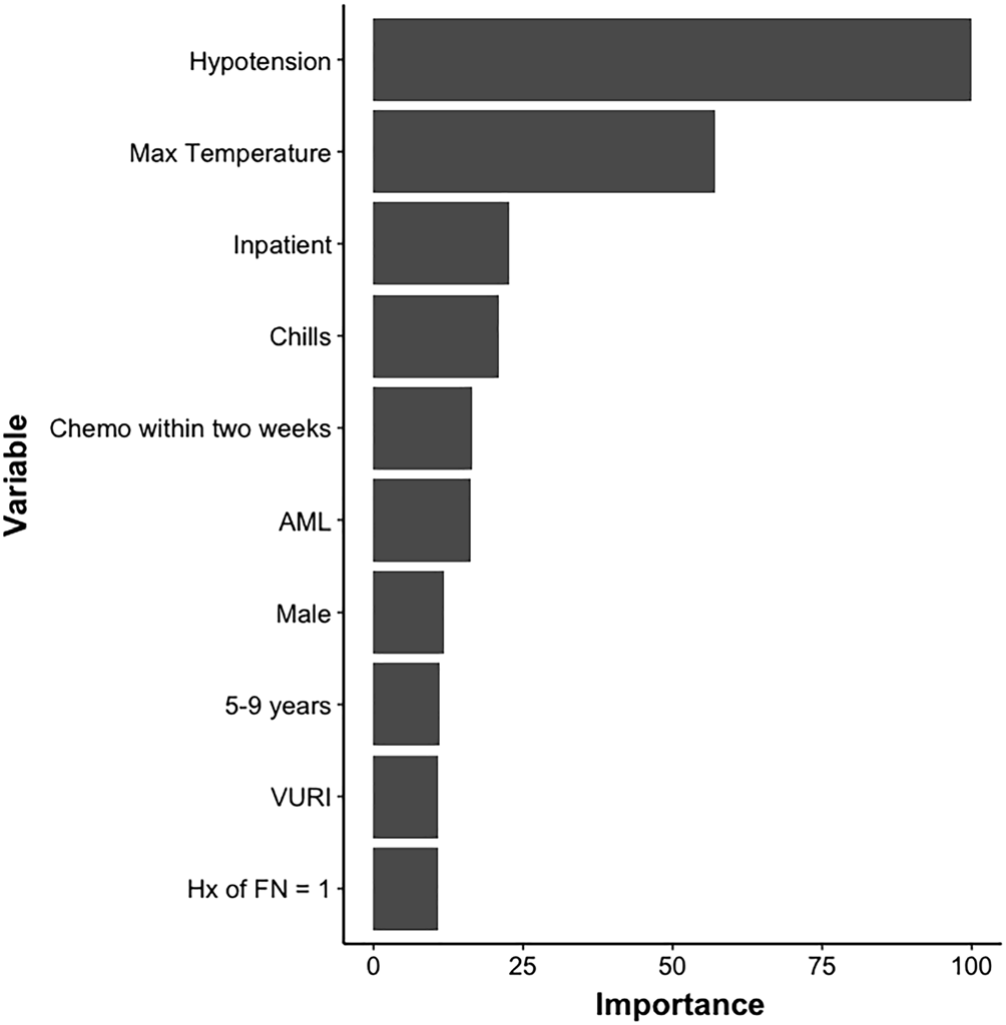
Depicts a list of variables important to the prediction of PICU admission with resampling of the multivariate analysis. The maximum temperature and hypotension recorded at time of presentation of FN episode were the most important variables for predicting transfer to PICU.

Frequency of variables and outcomes in the derivation and validation groups are shown in Table 2 (Fig. 2, 3).

**Table 2:** Characteristics, variables and outcomes in FNEs among derivation and validation cohorts (*N* = 505).

	All Episodes (*n* = 505) *N* (%)	Derivation cohort (2009–2014, *n* = 343 [68%])	Validation cohort (2015–2016), *n* = 162[32%])	*P* value
Age, mean (SD)	11.08 (6.4)	10.16 (5.8)	12.59 (6.8)	0.7
Female sex	107 (47)	159 (46)	69 (42)	0.8
Cancer diagnosis				
ALL/lymphoma	218(43%)	149 (44%)	69 (41%)	0.72
AML/mixed leukemia	89(18%)	54 (16%)	35 (21%)	0.42
Neuroblastoma	69(14%)	51 (15%)	18 (11%)	0.63
Other solid tumors	129(25%)	89 (26%)	40 (24%)	0.83
Neutropenia > 7 days prior FN	346 (68)	221 (70)	125 (75)	0.82
Prior BSI in last 12 months	133 (26)	83(24)	50 (30)	0.46
GI symptoms *n* (%)	173 (34)	125 (36)	48 (30)	0.52
Mucositis *n* (%)	111(22)	81 (24)	30 (18)	0.35
VURTI	167 (33)	109(31)	58 (35)	0.36
Chills	28 (6)	18 (5)	10 (6)	0.75
Hypotension	89 (18)	68 (20)	21 (13)	0.21
Temperature > 39 at presentation	122 (25)	91(26)	31 (19)	0.61
Chemotherapy in last 2 weeks	282 (76)	198 (57)	84 (52)	0.73
Inpatient (location FN)	85 (17)	52 (15)	33 (20)	0.42
Hx FN > 1	311 (61)	201 (58)	110 (68)	0.36
ALC < 300, *n* (%)	156 (31)	96 (28)	60 (37)	0.28
ANC < 100, *n* (%)	362 (72)	239(70)	123 (76)	0.52
AMC < 100 *n* (%)	389 (77)	251 (74)	138 (85)	0.19
Platelet < 50	308 (61)	203 (59)	105 (64)	0.26
Hb < 7	103 (20)	77 (22)	26 (16)	0.11
Prior GCSF	66 (13)	41 (12)	25(15)	0.24
BSI	106 (21)	66 (19)	40 (24)	0.31
Admitted to the PICU, *n* (%)	54 (11)	35 (10)	19 (12)	0.61

### Model performance

**Figure 4:**
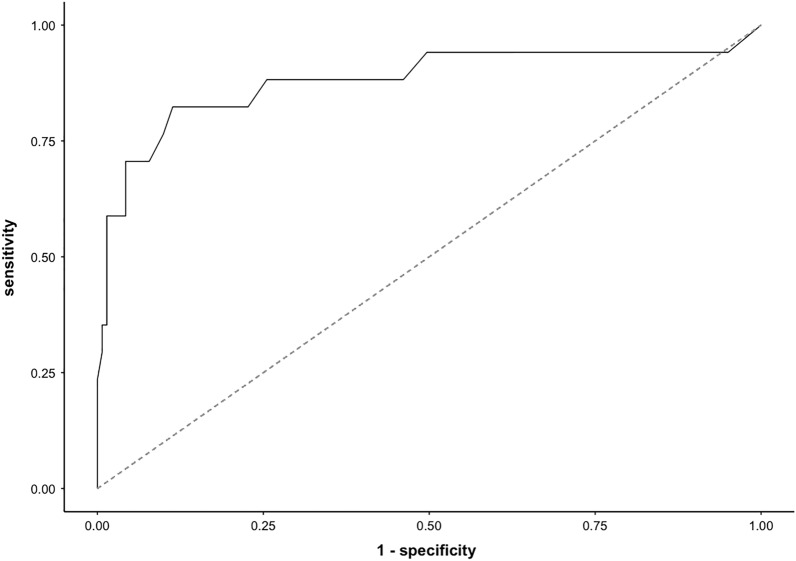
Receiver operating characteristic curve for random forest (RF) model in BSI prediction (AUC 0.79).

AUCs and statical performance for different prediction models using the prospective validation cohort are shown in Table 3. The logistic regression model (LReg) performed similarly to the Hakim model in predicting BSI (LReg AUC 0.65 vs. Hakim AUC 0.66), whereas the RF model predicted BSI much more accurately than the Hakim model (RF AUC 0.79 vs. Hakim AUC 0.66, *P* < 0.05) (Table 3) (Fig. 4). The RF model also performed best at predicting 14-day TIC as compared to the (LReg) model and the Hakim Model (RF AUC 0.88 vs LReg AUC 0.76, *P* < 0.05) and (RF AUC 0.88 vs Hakim AUC 0.65, *P* < 0.05).

**Table 3:** Statical Performance of 3 prediction models for BSI and TIC.

	Threshold	Sensitivity % (± 95%CI)	Specificity % (± 95%CI)	PPV % (± 95%CI)	NPV % (± 95%CI)	LR + (HR)	LR- (LR)	AUC (95% CI)
BSI								
Random Forest	0.056	81 (68–92)*	77 (65–89)*	51 (43–66)	91 (82–98)*	3.5*	0.24	0.79 (0.71–0.85)*
Logistic Regression	0.06	73 (63–84)	70 (61–80)	46 (55–38)	86 (78–90)	2.4	0.38	0.65 (0.53, 0.76)
Hakim	-	68 (62–74)	62 (54–71)	40 (36–48)	79 (71–87)	1.7	0.61	0.66 (0.56, 0.77)
TIC								
Random Forest	0.049	89 (78–97)*	87 (80–95)*	56 (47–64)	97 (92–99)*	6.8*	0.12*	0.88 (0.76, 0.99)*
Logistic Regression	0.053	81 (71–90)	83 (77–88)	46 (39–53)	92 (85–97)	4.7	0.22	0.76 (0.60, 0.92)
Hakim	-	71 (62–80)	72 (68–79)	35 (27–44)	80 (73–86)	2.5	0.4	0.65 (0.50, 0.80)

*P < 0.05 compared to the Hakim model.

For each outcome, a likelihood ratio (LR) was calculated with 95% credibility (post-test probability) to assess a probability of BSI or TIC and potential utility of prediction model in clinical practice The quantitative value of a calculated LR corresponds to the utility of a prediction tool to point towards or away from an outcome. LR+ is represented by a value 1 or greater, and the higher the value, the increasing ability to identify high risk patients. LR− is a value between 0 and 1, and the closer it is to 0, the better it can identify a low-risk patient. The RF model has a higher LR + compared with Hakim et al. in predicting high risk for BSI (3.5 vs 1.7, *P* < 0.05) and TIC (6.8 vs 2.6, *P* < 0.05), and a lower LR- in predicting low risk for both BSI and TIC (0.24 vs 0.6), and (0.12 vs 0.4) respectively. Based on these results, our model over performed Hakim et al. model in predicting FN outcomes in our cohort.

Because the RF utilizes a decision-tree type approach, the location of each variable across all trees is an approximate measure of the importance of that variable towards predicting the outcome. Figure 2 depicts the 10 variables most important to predicting BSI. Of these, temperature, low blood pressure, prior positive blood culture, and AML as underlying diagnosis are the most important variables used by the RF to predict positive blood culture. Temperature and low blood pressure are also highly important in the RF model for predicting TIC (Fig. 3).

## Discussion

In this study, we derived and validated a prediction model for BSI and transfer to ICU in a large, diverse population of children with cancer that demonstrated better performance than previously published methods. Independent risk factors for BSI and TIC included high temperature and low blood pressure on admission. The use of such risk factors to identify the patients who are at greatest risk for BSI and risk for TIC could help providers identify the appropriate level of care.

There is currently no single risk stratification system in widespread use in pediatric practice and there are considerable variations in practice. A simple, reliable, and safe risk stratification system will have potential to significantly reduce hospitalization rates and hospital length of stay without increasing overall mortality.

Several previous investigations of pediatric cancer patients with FN have identified different hematologic laboratory results^6,7,9,17,21^ (e.g., ANC, platelet count, or absolute monocyte count), clinical factors related to a patient’s underlying cancer (e.g., diagnosis of AML or the presence of uncontrolled relapsed disease) and vital sign abnormalities (e.g., fever, hypotension)^8,21–23^ as risk factors for BSI, MDI and adverse outcomes.

Most previous studies have treated temperature as a dichotomous variable (temperature > 39 or < 39 degrees)^8,12,17,22^. In our study, including maximal temperature as a continuous variable increased model performance in predicting BSI and TIC. We also identified hypotension as an independent risk for both BSI and TIC, like other studies^12,23^.

We identified prior BSI, regardless underlying diagnosis, as an additional variable important to predicting BSI. Subsequent BSI don’t represent relapse or inadequate treatment of a previous BSI, since subsequent BSI were caused by different pathogens than the index BSI. In addition, prior BSI remained a significant risk factor regardless of whether the central line was retained or removed. Interestingly, model performance doesn’t change even after exclude BSI caused by the common skin contaminant *Coagulase-negative* staphylococci (*CoNS*).

Similar to previous studies, we found that AML patients with FN are more likely to develop BSI compared with patients with other underlying diagnoses. This is not surprising since patients with AML receive more intensive chemotherapeutic regimens (i.e., Cytarbine containing regimen) than do other oncologic patients, leading to longer durations of neutropenia and thus a higher risk of infectious complications^24,25^. The rate of bacteremia within our cohort, 21%, is on the high end of the range reported in the pediatric FN literature^26,27^. We hypothesize that our limited use of antibacterial prophylaxis in high-risk FN patients could be contributing to this.

Strengths of our study include the relatively large patient population and number of events of the outcomes of interest (BSI and ICU transfer). Data from our data warehouse was supplemented by extensive manual review of the electronic medical record. We performed simultaneous assessment of potential risk factors allowing for the identification of independent factors predictive of BSI or ICU transfer, each of which we evaluated separately. Also, it is important to note that there are no current validated schemas for defining those patients at high risk of developing complications from FN. Our prediction model *n* not only identifies low risk, but also high-risk FN patients. Furthermore, most published prediction studies in FN include a have limited number of variables, while our study has analyzed a broader variety of elements including patient-specific factors (including age, malignancy type), treatment-specific factors (GCSF), episode-specific factors (including height of fever, hypotension, mucositis, blood counts).

The prediction and post-test probability of the model provides a robust method of determining pediatric cancer patients with high risk of BSI and transfer to ICU. The model performed better than other published models.

The current study has several limitations. First, it is a retrospective analysis at a single academic medical center and the results may not be generalizable to other institutions with different practices of antimicrobial prophylaxis and different empiric management of neutropenic fevers. While we did stratify patients into four groups based on cancer type, we did not look at the impact of chemotherapy phase and intensity (i.e. ALL) on outcomes. The retrospective nature of our study limited our ability to collect accurate objective clinical variables from the medical record such as mucositis, chills, and upper respiratory tract symptoms. Because overall mortality among our patients was low (2.3%), we lacked the power to perform subgroup analyses related to mortality as an outcome.

## Conclusion

In this study, we derived and validated prediction rules for BSI and transfer to ICU in pediatric cancer patients who have FN. Children and adolescents with higher fever and hypotension at presentation are at increased risk of BSI and transfer to ICU. Having a prior BSI is an additional risk factor for developing a subsequent BSI. To the best of our knowledge, our study is one of the few done in the United States in last decade assessing the risk factors predictive of an adverse outcome in pediatric patients with FN. The information gained from this study will help in formulating a risk prediction model that is easy to use, widely applicable, and clinically relevant. Prospective, external validation of this model is essential prior to implementation to risk stratify pediatric FN patients. After external validation, our next step to is to use this tool to facilitate antibiotic stewardship and early hospital discharge among pediatric cancer patients with FN.

## Supplementary Information

Supplementary Information.
