# Supplementary material for: A prediction model for bacteremia and transfer to intensive care in pediatric and adolescent cancer patients with febrile neutropenia
Source: Sci Rep. 2022 May 6;12:7429. doi: 10.1038/s41598-022-11576-z (PMC9076887; doi:10.1038/s41598-022-11576-z)
Supplement: Supplementary file 1 — Supplementary Information. [file 41598_2022_11576_MOESM1_ESM.docx]

Supplemental tables,

Supplementary Table 1: Full list of variables used in prediction of BSI and transfer to PICU

| Terms | Definitions |
| --- | --- |
| Fever (2) | A single oral temperature of >38.3ºC or temperature of >38.0ºC sustained over a 1-h period or on more than one occasion in a 24-hour period |
| Neutropenia(2) | An ANC <500 cells/microL or an ANC that is expected to decrease to <500 cells/microL over the next 48 hours |
| ANC | Total white blood cells multiplied by the percentage of neutrophils and bands on differential count |
| MDI(23) | Neutropenic fever with a clinical focus of infection and an associated pathogen (i.e., BSI, C. difficile infection [CDI], viral respiratory infection [VRI]) |
| Clinically documented infection (23) | Neutropenic fever with a clinical focus but without the isolation of an associated pathogen, (eg, cellulitis, pneumonia) |
| Unexplained fever(23) | Neutropenic fever with neither a clinical focus of infection nor an identified pathogen |
| True BSI(2) | Pathogen isolation from blood culture at time of presentation determined to be a pathogen (i.e., BSI) or a contaminant using the National Health Safety Network (NHSN) criteria for skin commensals and the clinical team’s decision to treat as a pathogen |
| Need for transfer for intensive care | All-FN episodes requiring PICU admission up to 14 days after diagnosis of FN. |
| Hypotension(24) | Blood pressure as a SBP or DBP below the fifth percentile for age |
| VURTI | Any upper respiratory symptoms at time of presentation confirmed with positivity of respiratory panel |
| Prior BSI | Any previous BSI whether during FN or non-FN episodes, and also include BSI from same line or different line |
| FNE count | Number of FN episodes before current FNEe, categorized to 3 groups: 0,1, or >1 episodes. Maximum FN episodes in cohort were 4 FNE per patients |

Supplementary Table 2. Variable’s categories

| Category | Variable list | Variable type |
| --- | --- | --- |
| Patient Demographics | : Age (years), Gender (male vs. female) | Categorical  Age (0-4 years,  5-9 years, >10 years)  Gender (male vs. female) |
| Patient History | Chemotherapy within two weeks, Prior positive blood culture, Count of FN episodes | Binary  Chemo within two weeks  Prior positive blood culture  Categorical  FN episode count (0, 1, more than 1) |
| Diagnosis | Cancer type (ALL+ Lymphoma vs. AML+Mixed Luekemia vs. Neuroblastoma vs. Other solid tumors) | Categorical |
| Location of FN | Inpatient vs. Outpatient | Binary |
| Clinical and Laboratory values | Maximum temperature indicating height of fever, low blood pressure, upper respiratory infection, chills, depth of neutropenia | Continuous  Height of fever  Binary  URI  Low blood pressure  Chills  Hemoglobin <7  Platelet <50  Prior G-CSF  ANC<100  AMC <100,  ALC <100,  Hemoglobin <7  Platelet <50,  Prior G-CSF |
| Medication (G-CSF) | Antibiotic administration | Binary |
